# The Selectivity of Molecularly Imprinted Polymers

**DOI:** 10.3390/polym13111781

**Published:** 2021-05-28

**Authors:** Gergely Becskereki, George Horvai, Blanka Tóth

**Affiliations:** Department of Inorganic and Analytical Chemistry, Budapest University of Technology and Economics, Szent Gellert ter 4., H-1111 Budapest, Hungary; becske3@gmail.com (G.B.); toth.blanka@vbk.bme.hu (B.T.)

**Keywords:** binding assay, HPLC, solid phase extraction, sensor, membrane, catalysis, QCM, potentiometric, voltammetric, optical

## Abstract

The general claim about novel molecularly imprinted polymers is that they are selective for their template or for another target compound. This claim is usually proved by some kind of experiment, in which a performance parameter of the imprinted polymer is shown to be better towards its template than towards interferents. A closer look at such experiments shows, however, that different experiments may differ substantially in what they tell about the same imprinted polymer’s selectivity. Following a short general discussion of selectivity concepts, the selectivity of imprinted polymers is analyzed in batch adsorption, binding assays, chromatography, solid phase extraction, sensors, membranes, and catalysts. A number of examples show the problems arising with each type of application. Suggestions for practical method design are provided.

## 1. Introduction

Molecular imprinting has turned into a broad research area in the last decades [1,2,3,4,5]. There are around 1000 papers published on molecularly imprinted polymers (MIPs) every year, and this figure appears to exceed the publication intensity of many other fields. The prime argument for making so many MIPs has been that MIPs can interact with their respective templates rather selectively, so that they have the potential to be used almost everywhere in chemistry, be it for analytical, environmental, catalytic or other technical applications. Early research with MIPs focused on small molecules as templates, and on simple polymer structures. Lately, MIPs have also been prepared for proteins and microorganisms, usually with surface imprinting. MIPs in nano size and/or enhanced by coupling with nanomaterials such as graphene oxide, carbon nanotubes or other nanoparticles, have also become common.

As the selectivity of MIPs is attributed to the creation of imprinted binding sites, it would be useful to know the chemical structure of these sites. However, despite the success in the preparation and applications of MIPs, there is relatively little information about the chemical structure of the binding sites unique to each MIP. Computer modeling of MIPs has a great potential in this respect [6], but direct experimental evidence to support the calculated binding site structures appears to be scarce. Adsorption experiments with MIPs have led many researchers to the conclusion, that most MIPs, and particularly the non-covalently imprinted ones (which represent the majority of MIPs) have heterogeneous binding sites. Some of these sites bind the template more strongly than others. The stronger sites are also regarded to be more selective towards the template than the weaker ones. Using this model for selectivity appraisal has its difficulties, as will be pointed out below.

Lacking a firm mechanistic base for explaining MIP selectivity, one may treat selectivity phenomenologically. This may seem to be an easy task: one needs merely to compare the behavior of the MIP towards its template and an interfering compound, respectively. This comparison should afford a figure, which can characterize the selectivity of the MIP against the interferent. The figure may depend, of course, on the composition of the medium where the MIP is used, and on other obvious parameters, such as temperature, but when these conditions are fixed, the selectivity figure should characterize the MIP’s selectivity unambiguously.

The present study shows that the concept of MIP selectivity is not so simple, and that the selectivity of the same MIP (in the same medium, at the same temperature, etc.) depends on the particular experiment and the application for which the MIP is employed. Typical applications of MIPs will be presented, and the differences in the concept and in the characterization of selectivity in these domains will be demonstrated.

## 2. Definition and Quantitative Characterization of Selectivity

Appreciation of MIP selectivity demands that one first defines selectivity in general. Chemists lean to think that selectivity has a simple definition, but a survey of the relevant literature [7] shows that this is not the case. IUPAC committees have tried to define selectivity in analytical chemistry [8,9], with almost no definite outcome. The ultimate degree of selectivity, called “specificity” by IUPAC, means basically, that an analytical determination accurately returns the analyte concentration of any sample. In real life, this strict condition can be relaxed to requiring accuracy merely within some prescribed tolerance limits. Even so, such near complete selectivity exists only in theory. In practical life, analytical chemists make some sort of educated guess about the expected error due to interferences. One way to do this is to limit the allowed range of samples (e.g., to “bovine milk”, or to a particular type of waste-water, etc.), and in this range of allowed samples the method may be regarded specific.

The situation is different when a new (analytical) tool, such as a MIP, is still in the development phase. At this stage, the developers may only want to map the potential interferences of the future applications. So, they may be satisfied by investigating the likely interferents one by one, and at first only in simple, artificial solutions. Such preliminary studies are permissible at an early phase of method development, but they may be misleading, as will be shown later. In the preliminary study, one would measure at first the concentration dependence of some quantity, e.g., of a distribution coefficient or of the signal of a MIP sensor, in separate solutions of the analyte and of each interferent, respectively. This may be followed by studying mixtures of the analyte with interferent(s), in different combinations. However, even if one takes only a few interferents, and only a few concentration levels for each of them, the number of experiments, to be carried out in the mixtures, will be very high. The mathematical tool called “design of experiments” may be helpful, but it is seldom employed with MIPs to discover their selectivity pattern.

Alternatively, one may try to confirm some expected mathematical relationship between the measured quantity (e.g., a signal) and the concentrations. This route had been taken for instance in potentiometry, with ion-selective electrodes [10,11]. Surprisingly, even if the mathematical form of the relationship between the signal and the concentrations is known, there may not exist a meaningful definition of a unique number, which quantitatively measures the selectivity for the target compound against an interferent. This will be explained in the next section.

MIPs are often used as part of a more complex system (e.g., in a sensor), where other elements of the system may have their own selectivities. For example, in an optical MIP sensor, the light absorption at a given wavelength may be the measured quantity. This signal is already by itself selective for compounds absorbing at that wavelength. The overall selectivity observed is the combined result of MIP selectivity and the optical “transducer’s” selectivity.

The ultimate test of the selectivity of a MIP method is the practical testing in the intended application, under a large variety of the possible conditions. In the case of analytical applications, this means the comparative analysis of many different samples with the MIP method compared to a standard method. If one wishes to appreciate the difficulty of achieving real life selectivity, one needs only to read recent papers on the unreliability of the selectivity of antibodies [12,13,14].

### 2.1. The Easy Case: Quantitation of Selectivity, If the Concentration Dependence of a Signal Measured in Mixed Solutions Is (Pseudo)Linear

In certain subfields of (analytical) chemistry, researchers seem to have good definitions for selectivity, despite the difficulties mentioned above. Examples for this may be found with many electroanalytical and spectroscopic methods. The basis for a well-defined quantitation of selectivity in these methods is the (pseudo)linear relationship [15] between the measured signal, and the concentrations of the analyte and the interferents, respectively. Expressed in a simple form, this relationship means that:

(1) $$Y=f\left(k_{A}\times c_{A}+k_{B}\times c_{B}+k_{C}\times c_{C}+\cdots \right),$$

where *Y* is the measured signal, *c_A_* is the analyte concentration, *c_B_* and *c_C_* are interferent concentrations, and the k-s are non-negative constants. If this relationship holds, selectivity for A against B can be expressed by a selectivity coefficient, *S_AB_*:

(2) $$S_{AB}=\frac{k_{A}}{k_{B}}.$$

One should note, that in Equation (1) the influence of any component’s concentration on the signal *Y* is independent from the effect of all the other components’ concentrations. An important consequence of this is that the separate “calibration” curves for compounds A, B, C, etc., provide all the needed information to express selectivity against the various interferents. Additionally, note that a special case of the pseudo-linear relationship is the simple linear additive relationship between the signal and the concentrations.

Transformation of Equation (1) shows the meaning and the utility of selectivity coefficients:

(3) $$Y=f\left(k_{A}\times \left[c_{A}+\frac{c_{B}}{S_{AB}}+\frac{c_{C}}{S_{AC}}+\ldots \right]\right).$$

This expression shows that the effect of *c_B_* on the signal is equivalent to replacing compound B with a concentration increase of A by Δ*c_A_* = *c_B_*/*S_AB_*. An important consequence of this is, that the analytical error due to the presence of interferents is *c_B_*/*S_AB_* + *c_C_*/*S_AC_* + ….

The relative error in the determination of A, in the presence of interferents, is then:

(4) $$\frac{error\;of\;\left(c_{A}\right)}{c_{A}}=\frac{\frac{c_{B}}{c_{A}}}{S_{AB}}+\frac{\frac{c_{C}}{c_{A}}}{S_{AC}}+\cdots .$$

### 2.2. The Difficult Case: Selectivity If the Concentration Dependence of the Measured Signal Is Not (Pseudo)Linear

When the concentration dependence of the measured signal is not (pseudo)linear, then Equation (3) is not valid. This precludes the possibility of expressing selectivity with unique figures for each individual interferent. So, simplicity is lost, and ambiguity may arise. This may lead then to some confusion in the assessment of the selectivity of an (analytical or other) tool, such as a MIP, especially if the same MIP is used in different applications. This ambiguity will indeed be seen in the following sections, where specific applications and examples will be discussed.

There are many analytical applications where only small interference effects need to be studied. For example, when using a sensor, measurement errors larger than, say, 10% may not be acceptable. Thus, one needs to study only how such small errors arise, as compared to interferent-free samples. It may be possible then, to approximate the signal deviations (or the analytical error) by linear additive functions. The coefficients of this function (and consequently the selectivity) might, however, depend on the analyte concentration in the sample.

The respective effects of different compounds on the signal may be independent and additive, even if a (pseudo)linear relationship does not hold. In form of functions:

(5) $$Y=f\left(g_{A}(c_{A}\right)+g_{B}\left(c_{B}\right)+g_{C}\left(c_{C}\right)+\cdots ),$$

where the indexed *g*–s are univariate functions of the respective concentrations, and the function *f* depends on the individual concentrations only through the g functions. For example, in the case of MIPs, the MIP might have separate, perfectly selective binding sites for each compound. In this case, the *g* functions could be the independent, separately measurable isotherms of the individual compounds. Some output function *Y*, such as the total adsorbed mass on the MIP, might be calculated then from the individual measurements of each compound. A constant selectivity would not exist, however, unless the (pseudo)linear relationship (which is a special case of Equation (5)) were to be valid.

## 3. Selectivity in Various MIP Applications

### 3.1. General Remarks

When a novel MIP is made for a template A, the typical statement made about the MIP is that it is selective for A against some similar compounds, and against most of the less similar compounds. To prove this, experiments are carried out. In many MIP applications, the result of the experiment (e.g., a signal) is not a (pseudo)linear function of the concentrations. As a result, there is no simple and unique way to express the MIP’s selectivity quantitatively.

Qualitative statements about MIP selectivity are also not easy to interpret. Some authors may call a MIP selective if it shows any preference for the template, be it even a small one. Others may then regard a MIP selective only if it is nearly specific for the template in a particular experiment.

In the next sections, some of the important applications of MIPs will be discussed and compared for their selectivity related features.

### 3.2. Batch (Static) Adsorption

**Figure 1:**
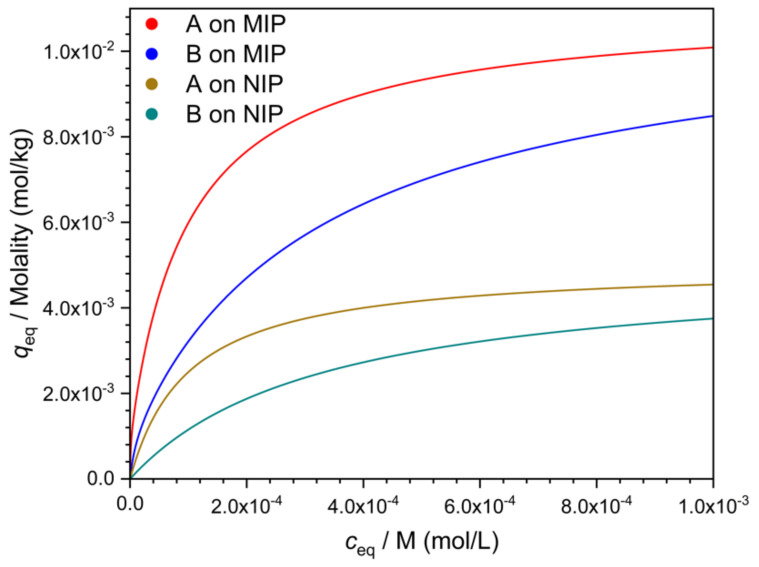
Adsorption isotherms of the template (**A**) and of an interfering compound (**B**) on the MIP and the NIP, respectively. *c_eq_* and *q_eq_* are the respective equilibrium concentrations in the solution and on the polymer. The curves were constructed with the bi-Langmuir model (see Section 3.2.1) for the MIP, and with the Langmuir model for the NIP. Binding capacity of the strong sites of the MIP (site No. 1): *q_s1_* = 10^−3^ mol/kg, of the weak sites of the MIP (site No. 2): *q_s2_* =10^−2^ mol/kg. Binding capacity of the NIP sites: *q_sNIP_* = 5 × 10^−3^ mol/kg. Binding constants on the MIP: K_A1_ = 10^6^ M^−1^, K_A2_ = 10^4^ M^−1^, K_B1_ = 10^5^ M^−1^, K_B2_ = 3 × 10^3^ M^−1^. Binding constants on the NIP: K_ANIP_ = 10^4^ M^−1^, K_BNIP_ = 3 × 10^3^ M^−1^.

Equilibrium adsorption of the template from a mixture is rarely useful as a separation method, but adsorption data provide some basic information about the MIP, and therefore batch adsorption measurements are presented in many MIP studies. In a typical measurement [16], different concentrations of the template in a suitable solvent are equilibrated with the MIP, the equilibrium solution concentration is measured, and an adsorption isotherm, similar to the one in Figure 1, is constructed. The same experiment is also done with a control polymer (usually with a nonimprinted control polymer, the NIP) (Figure 1). Similar measurements are also made with the likely interfering compounds (Figure 1). The isotherms are usually nonlinear. The vertical distance between the MIP and NIP isotherms of the template (A) is sometimes called “specific binding”.

#### 3.2.1. Batch Adsorption in Separate Solutions

**Figure 2:**
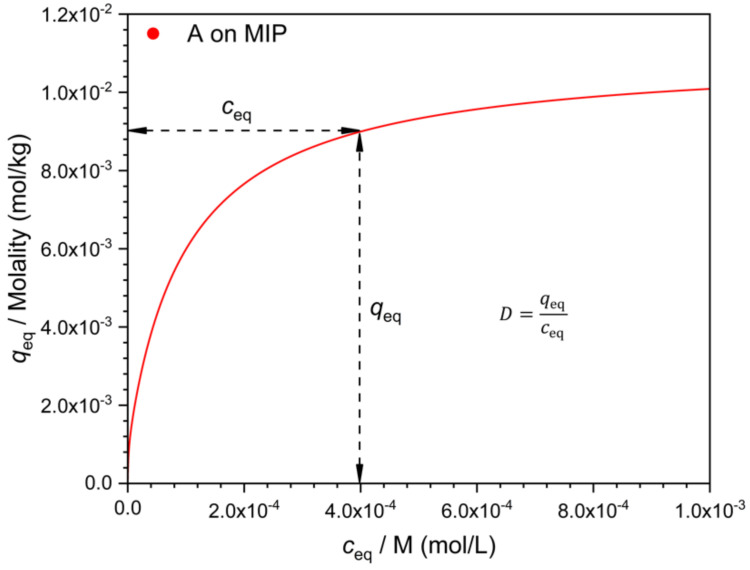
Determination of the distribution coefficient *D* from the isotherm, exemplified for the template (**A**) on the MIP. Parameters of the isotherm as in Figure 1.

**Figure 3:**
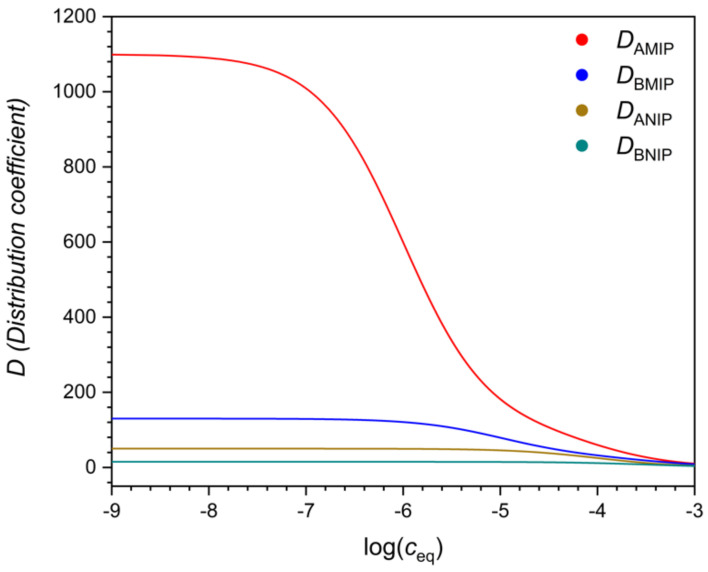
Distribution coefficients *D*, as the function of the logarithmic equilibrium solution concentrations, calculated for the isotherms shown in Figure 1.

**Figure 4:**
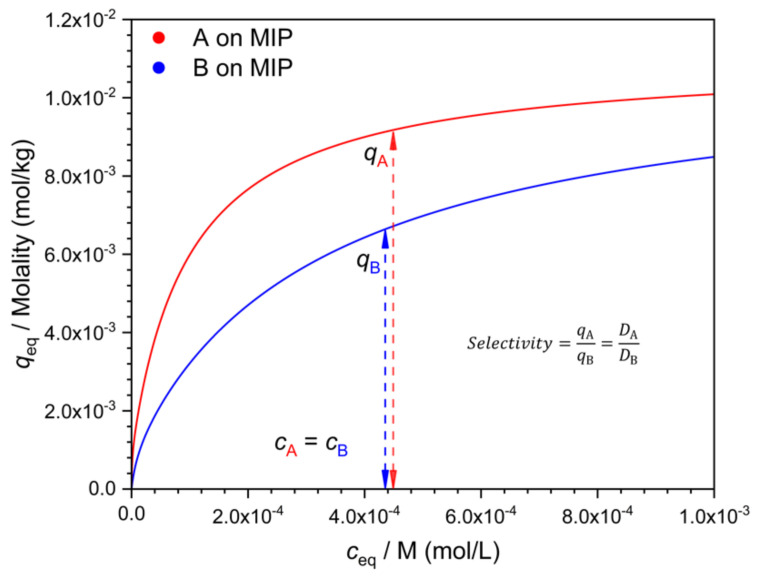
Determination of the selectivity from separately measured isotherms of the template (**A**), and the interferent (**B**), using the respective *q* values measured at identical *c* values. Parameters of the isotherms as in Figure 1.

There are many possible ways to characterize selectivity from such data, and as will be seen, they are not all equivalent. One may compare the adsorbed concentration of the template (*q_A_*) to that of the interferent (*q_B_*), determined at the same solution concentration value, i.e., at *c_A_* = *c_B_* (Figure 2). The ratio *q*/*c* gives the distribution coefficient *D*. The ratio *q_A_/q_B_* = *D_A_*/*D_B_* is a measure of selectivity. Due to the nonlinearity of the isotherm, the *D* values (*D_A_* and *D_B_*) are concentration dependent (Figure 3). Their ratio, the selectivity (Figure 4), may also depend on the concentration, but this is not necessarily the case.

**Figure 5:**
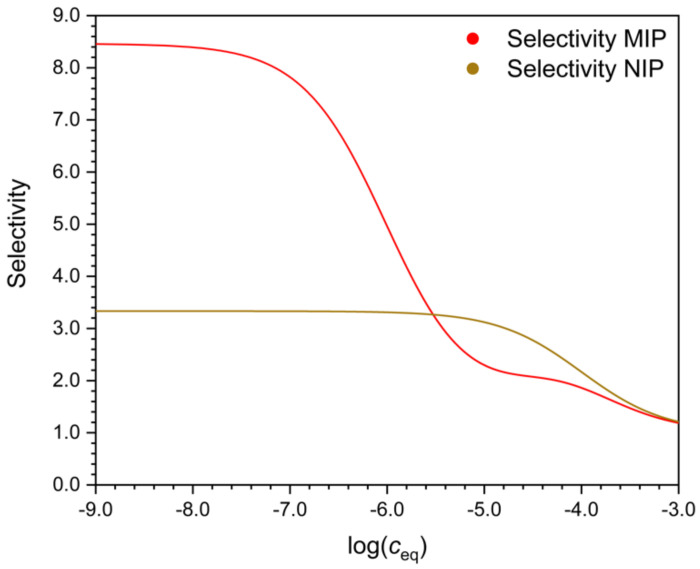
Selectivity of the MIP and the NIP, respectively, as a function of the logarithmic equilibrium solution concentration. Parameters of the curves as in Figure 1. Selectivity is calculated as shown in Figure 4.

The non-imprinted control polymer (NIP) may show selectivity for either the template A, or the interferent B. The selectivity of the NIP can be measured also as the ratio of the respective *q*-s or *D*-s at the same *c* as for the MIP. The selectivity of the MIP may be compared to that of the NIP by calculating the ratio of their respective selectivities, at the same *c* value. This ratio, i.e.,

(6) $$\frac{\frac{q_{AMIP}}{q_{BMIP}}}{\frac{q_{ANIP}}{q_{BNIP}}}=\frac{\frac{D_{AMIP}}{D_{BMIP}}}{\frac{D_{ANIP}}{D_{BNIP}}}=\frac{IF_{A}}{IF_{B}},$$

where *IF* = *D_MIP_*/*D_NIP_* is the “imprinting factor” of the respective compound, reflects the improvement in selectivity due to imprinting. Imprinting typically increases the binding of the template (*D_AMIP_* > *_ANIP_*), and it also increases the selectivity (*D_AMIP_*/*D_BMIP_* > *D_ANIP_*/*D_BNIP_*). Sometimes, however, the selectivity of the MIP against an interferent is not better than the selectivity of the NIP [17]. Figure 5 shows that there may exist a concentration range, where the selectivity of the NIP is better than that of the MIP, whereas in other concentration ranges the MIP is more selective. The reason of this peculiar behavior will be explained later.

**Figure 6:**
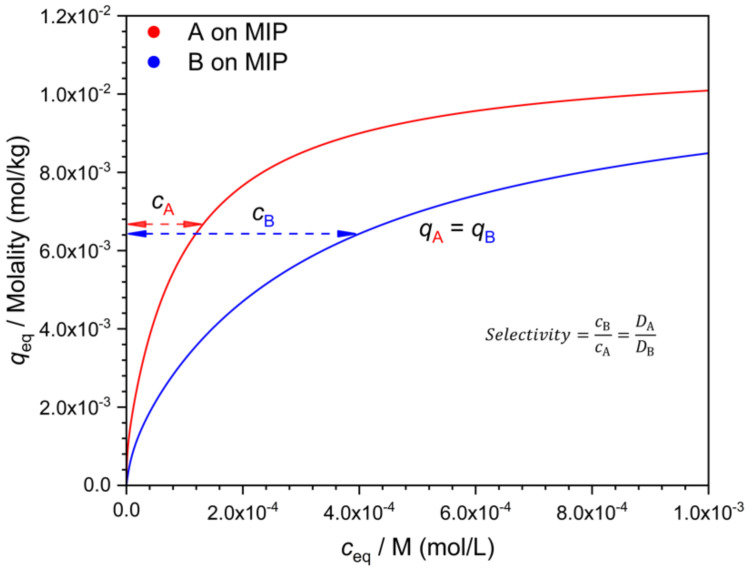
Determination of the selectivity from separately measured isotherms of the template (**A**), and the interferent (**B**), using the respective *c* values measured at identical *q* values. Parameters of the isotherms as in Figure 1.

An alternative to the comparison of binding (*q*) at equal *c* values, is the comparison of the respective *c* values at identical *q* values (Figure 6). Indeed, there is little reason to prefer either choice. The numerical values, however, depend on this choice when the isotherms are nonlinear. A third, more general way of calculating selectivities from individual isotherms has been presented by Ansell [16].

A completely different approach to selectivity consists of fitting a mathematical model to the individual isotherms of the template A and the interferent B, respectively [16]. There exist many isotherm equations, which have been fitted to MIP isotherms, and an especially often used one is the bi-Langmuir isotherm. The physical model behind this isotherm assumes the existence of two separate, independent types of binding sites on the MIP. The respective binding site concentrations, and their respective binding strengths, can be obtained by fitting the bi-Langmuir equation, Equation (7), to the measured isotherm points [18].

(7) $$q_{A}=\frac{q_{s1}\times K_{A1}\times c_{A}}{\left(1+K_{A1}\times c_{A}\right)}+\frac{q_{s2}\times K_{A2}\times c_{A}}{\left(1+K_{A2}\times c_{A}\right)},$$

where *q_s_*_1_ and *q_s_*_2_, respectively, denote the concentrations of the two binding sites on the polymer. *K*_*A*1_ and *K*_*A*2_ are the respective binding constants of the compound A on the two sites. With MIPs, one may often observe large differences in the parameters (*q_s_* and *K*, respectively) of the two kinds of sites. One type of site is abundant but rather weak, while the other type is scarce but strong. The NIP often appears to have only one kind of site, which is abundant but weak. These observations have resulted in a model where the weak sites of the MIP behave essentially the same way as the NIP sites, while the strong sites of the MIP are the result of imprinting. This model leads to the conclusion that the strong sites of the MIP are also responsible for the increased selectivity of the MIP against the NIP. Hence, in this model, selectivity may apparently be characterized by subtracting the NIP isotherm from the MIP isotherm, and taking the resulting difference curve (“specific binding”) as the concentration dependent selectivity measure (Figure 1). This approach is better avoided, however, if the underlying model has not been proved. One reason for the invalidity of this model may be that imprinting may also increase the number of accessible non-imprinted sites. The figures in this paper were plotted with the latter assumption.

If the MIP follows the bi-Langmuir model, and the strong sites are indeed more selective than the non-imprinted sites, then the selectivity of the MIP may still become inferior to the NIP in a certain concentration range, as shown in Figure 5. The reason for this is, that for any Langmuir type site, the distribution coefficient increases markedly around the concentration 1/*K*, where *K* is the equilibrium constant of the site. The selectivity (of the strong binding sites) for A means that *K_A_* is higher than *K_B_* on the stronger sites. Therefore, as one moves to lower concentrations, the distribution coefficient of B, *D_B_*, begins to increase sooner than *D_A_*. There will be a concentration range where the relative increase of *D_B_* is faster than of *D_A_*, and the selectivity of the MIP for A may increase more slowly than the selectivity of the NIP.

Note here, that as Figure 5 shows, the selectivity of MIPs often increases greatly at very low solution concentrations but may be low at high solution concentrations. For this reason, a MIP that is found to be very selective in a binding assay, may have poor selectivity in a sensor or a technical separation, employed at higher concentration levels.

Another possibility to characterize selectivity, still within the bi-Langmuir model, is to compare the binding constant of the template on the strong sites to the binding constant of the interferent on the same sites.

The above considerations show that even from the same experimental isotherms one may arrive at different selectivity measures of a MIP. It is of further note that alternative isotherm models to the Langmuirian have also been used with MIPs [16], and these appear to be even more difficult to be used for selectivity estimates.

#### 3.2.2. Batch Adsorption from Mixtures

The selectivity measures described above have been based on measurements in separate solutions of the template and the interferent, respectively. In any real application, however, these compounds would be found together in the samples. If the two compounds were adsorbed from their mixture independently from each other, then one might rely on their individual isotherms, measured separately. However, this occurs rarely with MIPs. The template and the interferent usually compete for the binding sites, while sometimes one of them assists the other’s adsorption. There is apparently no general rule to predict what will happen, let alone to calculate some measure of selectivity. Occasionally the competitive Langmuir or bi-Langmuir equations [18] may be valid, or some other, empirical relation may be found [19]. To prove such relations, many experiments may be necessary, which have been rarely done with MIPs. It is educative, in any case, that if the simple competitive Langmuir model (with one kind of site) is valid, i.e.,

(8) $$q_{A}=\frac{q_{s}\times K_{A}\times c_{A}}{\left(1+K_{A}\times c_{A}+K_{B}\times c_{B}\right)},$$

(9) $$q_{B}=\frac{q_{s}\times K_{B}\times c_{B}}{\left(1+K_{A}\times c_{A}+K_{B}\times c_{B}\right)},$$

then

(10) $$\frac{D_{A}}{D_{B}}=\frac{\frac{q_{A}}{c_{A}}}{\frac{q_{B}}{c_{B}}}=\frac{K_{A}}{K_{B}}.$$

This means that *D_A_*/*D_B_* is a constant, and equal to the ratio of the respective equilibrium constants, i.e., to *K_A_*/*K_B_*, independently from the concentrations of the two species.

This result is very different from the situation discussed above, where the selectivity was derived by the comparison of the individual isotherms, as in Figure 4 or Figure 6. One consequence of this difference is that in mixed solutions (if the competitive mono-Langmuir model is valid) the selectivity does not decrease at higher concentrations. On the other hand, the amounts adsorbed from each compound are less, in the case of competition, than from the separate solutions.

### 3.3. Binding Assays

One of the early great successes of MIPs was the development of the theophylline binding assay using a MIP imprinted with theophylline [20]. This assay is a close analog of antibody binding assays. The theophylline selective (strong and scarce) binding sites of the MIP act similar to immobilized antibodies. Radioactive theophylline is used as a tracer. The tracer competes for the binding sites with the non-radioactive theophylline in the assayed sample. The binding assay relies on the nonlinearity of the theophylline binding isotherm of the MIP [21]. Many other MIP binding assays have been developed since the cited pioneering work [22,23,24].

**Figure 7:**
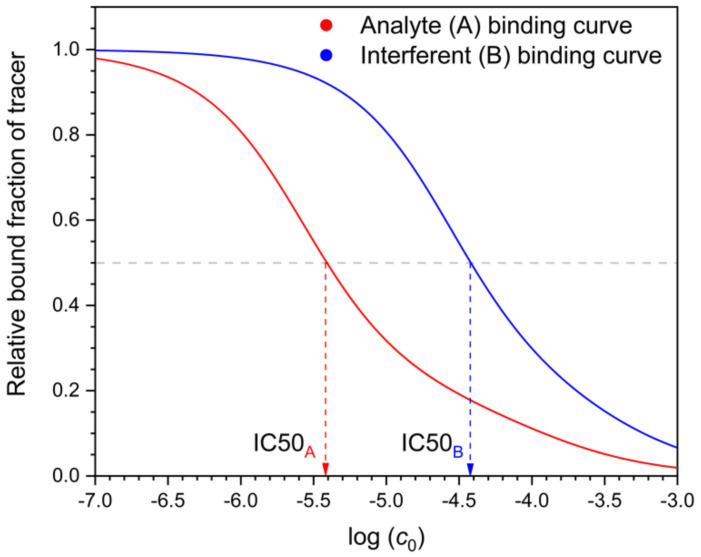
Binding curves and IC50 values of the analyte (**A**) and an interferent (**B**) on a MIP. Parameters of the curves, as shown in Figure 1. The tracer is radioactive A, and its concentration is negligible compared to the non-radioactive A concentrations. *c_0_* is the solution concentration of A or B before equilibration.

The selectivity of MIP binding assays has been characterized analogously to immunoassays, by measuring the IC50 values of the template and the individual interferents, respectively [16,20]. IC50 values are obtained from displacement curves of the tracer by the template (analyte) and the interferent, respectively (Figure 7). In the case of the interferent, B, the measurement occurs in mixed solutions of radioactive (“hot”) A (the tracer) and the “cold” interferent, respectively. One might think that this is an ideal method for measuring selectivity because it occurs in a mixed solution. The measurement shows essentially the change of the tracer A’s distribution coefficient as the function of the interferent (B) concentration. Yet this is being done only at a single, extremely low concentration of the tracer A. From these results alone, one cannot calculate the effect of the interferent on the analyte A’s distribution at realistic analyte concentrations, because the latter are orders of magnitude above the tracer concentration. To assess the selectivity under realistic conditions from the IC50 values, one needs to know also the equations describing the adsorption of the two compounds in mixed solutions. For immunoassays (using antibodies, not MIPs), equations corresponding to the competitive Langmuir isotherm have been used as a crude approximation [25]. For MIPs, the existence of this problem concerning the need of a valid model has apparently not been adequately recognized. It would therefore seem to be a good practice in MIP binding assays, to study, at different realistic analyte concentration levels, how the addition of the interferent biases the measured analyte concentration. Such practice has been recommended for the analogous biochemical ligand binding assays by a committee of the American Association of Pharmaceutical Scientists [26].

It is interesting to note that when the IC50 value of the interferent is measured, one does not measure the distribution of the interferent between the solution and the MIP, because only the tracer (radioactive analyte) distribution is measured. Thus, the binding assay selectivity measure does not involve any direct information about the binding of the interferent relative to the analyte.

MIP binding assays have been realized as well with non-radioactive tracers. The displacement of a colored or fluorescent tracer is similar to the displacement of a radioactive tracer, but the tracer and the analyte are not identical chemically in this case. This makes discussion of selectivity even more complex, but the same problems that were observed above with homologous radiotracers remain.

### 3.4. HPLC

MIPs have been used as stationary phases in liquid chromatography [27]. They have proved particularly useful in separating enantiomers. MIP HPLC measurements have also been used in fundamental studies of MIP selectivity [28,29]. The selectivity is typically expressed by the ratio of the retention factors of analyte and interferent, respectively. The use of retention factors for substances with non-linear adsorption isotherms (as typical with MIPs) is very problematic [18,30]. Notwithstanding this, the qualitative picture emerging about the chemistry of imprinting from such selectivity studies has proved useful.

When MIP HPLC is used for real life separations, one needs to recognize that the ratio of retention factors is not an analytical selectivity measure, not even in linear chromatography. To stress this difference, IUPAC requires to call this ratio the separation factor, and not selectivity [31]. Indeed, a separation is selective in the analytical sense if the measured peak area (or peak height) of the analyte is not modified by the interferent. This is achieved if the column perfectly separates the analyte from the interferent. For this, one needs to consider not only the retention factors, but also the peak dispersions. MIP columns are usually plagued by broad and tailing peaks, and this may limit the chromatographic selectivity of MIP columns, despite good separation factors.

If the separation factor is sufficiently large, say about 2, one may obtain good separation even on MIP columns. A separation factor of 2 translates to *D_A_*/*D_B_* = 2, at least in the case of linear chromatography. This is, however, a rather low (practically useless) selectivity for the separation of the two compounds in batch adsorption. In a QCM sensor, this same level of selectivity would be also very poor (see below). Since many novel MIPs had been tested for their selectivity as HPLC column packings, statements about an observed “high selectivity” of the MIP must be carefully interpreted.

It is tempting to believe that the selectivity of a MIP, as calculated from retention factors measured with a MIP column, is the same as the selectivity obtained by comparing distribution coefficients measured by equilibrium batch adsorption. One should not forget, however, that in nonlinear chromatography such a relationship is not valid [18]. Moreover, the contact time of the sample zones with the MIP particles in the HPLC column may be too short for equilibration.

An interesting question is how competitive adsorption may influence MIP HPLC. In preparative liquid chromatography [18] competition is an important phenomenon. It leads to peak distortions and to shifts in retention. With MIPs, such effects have rarely been noted in chromatographic experiments, although competitive adsorption is in fact the basis of other MIP applications, such as binding assays.

### 3.5. SPE

Solid phase extraction (SPE) is a practically very relevant application of MIPs [27]. With other, more conventional sorbents, SPE serves mainly for group separations, similar to the separation of hydrophobic components of a sample from hydrophilic ones. The special selectivity patterns of MIPs afford separations different from the conventional sorbents.

SPE may be considered as a chromatographic system with a very low plate number. Thus, for meaningful separations, one needs high separation factors, i.e., substantial differences between the binding of the analyte(s) and the other components of the sample, respectively. Some MIPs bind their respective templates very strongly even when compared to substances similar to the template. However, quite often, these differences are not sufficient for separating the template from similar substances on an SPE cartridge (offline or online). The majority of MISPE (i.e., MIP SPE) procedures serve only to separate the template, together with its close analogs, from chemically quite different substances. The goal is to remove from the sample those unrelated compounds, which would incidentally coelute with the analyte(s) from the HPLC column. The task of separating the chemically closely related compounds, which are all retained on the MIP column, is well solved by the HPLC system itself. In conclusion, MISPE is mainly, similar to other SPE methods, a group separation technique, but with different selectivity pattern from other SPE sorbents.

### 3.6. Sensors

The selectivity requirements towards MIPs are very high in sensor applications [32,33]. Sensors are usually employed in the unmodified sample, and thus potential interferents are not removed by sample pretreatment. If the goal is to determine the analyte in the presence of analogous compounds at comparable concentrations, the selectivity must be very high. Few conventional MIPs can satisfy this requirement. It seems that in many cases, where MIP sensors are indeed very selective against analogs, the mechanism of detection is not perfectly clear yet.

There is a vast literature on MIP sensors and sensor arrays. Authors of a recent review [34] have found 2500 publications on MIP sensors, of which 18.5% had been published in the single year before the review was written.

Authors frequently assert without convincing proof that the selectivity of the sensor is due to imprinting. The difficulties of interpreting the selectivity of MIP sensors will be illuminated by the example of a good analytical paper [35]. This work presents a useful method for the determination of phenol in waters. An optical sensor was constructed using a sensor membrane incorporating a MIP for phenol. After equilibrating the membrane with the sample, a conventional color reaction for phenol detection was employed. This caused the membrane to absorb visible light. The absorbance was proportional to phenol concentration. Analysis of real samples with this sensor gave identical values to those obtained with the same color reaction in homogeneous phase. Consequently, this was a very successful sensor. Yet, as far as one can judge, the selectivity of this MIP method relied on the selectivity of the color reaction, although the authors claimed that it was due to the MIP. This contradiction appears to be due to the measure of MIP selectivity, applied by the authors, which was the difference between the phenol binding of the MIP and the NIP, respectively, i.e., *q_MIP_* − *q_NIP_* at the same *c* (i.e., the “specific binding”). This difference was indeed substantial, although in the actual case it seems to have had little to do with the sensor’s overall selectivity. The example shows that depending on one’s definition of selectivity, very different assessments may be given for the same experimental results.

Due to the complexity and the fast development of the MIP sensor field, it cannot be attempted yet to make general statements about the selectivity of MIP sensors. Therefore, only some short notes will be made about the common sensor types. Examples will also be shown to illustrate the difficulties of interpreting sensor selectivity. The particular examples were selected because they had received good notes for their selectivity in recent reviews. As will be seen below, the most frequent problems with MIP sensor selectivity measurements are:

- Lack of measurements in mixed solutions of analyte and interferent(s)
- Measurement of selectivity only at a single concentration
- Disregarding that the nonlinear sensor response to the analyte makes interpretation of selectivity measurements difficult
- Little information is given about the likely interferences and their levels in real samples
- “Real samples” are mostly restricted to a single, oversimplified case.

#### 3.6.1. QCM Sensors

The case of the quartz crystal microbalance (QCM) sensors is apparently the simplest. The MIP is employed as a thin layer on the sensor electrode. Adsorption of any compound from the sample changes the mass of the MIP layer, and this is detected as a frequency shift. If two compounds are simultaneously adsorbed, the mass change is the sum of the two adsorbed masses. Selectivity may be described as in the case of batch adsorption from mixtures. However, in contrast to the usual molar adsorbed concentrations, *q*, the mass concentrations (M × *q*, where M is the molar mass) are relevant in the case of QCM sensors. In other words, the molar masses of the analyte and the interferent, respectively, do also influence selectivity.

In one of the many MIP QCM sensor papers, Battal et al. [36] prepared a sensor for various synthetic cannabinoids. As noted in a favorable review on this work [32], the measurements in separate solutions of closely related compounds (at equal concentrations) showed marked differences in the signals. Unusually high selectivities were observed between large heterocyclic molecules differing only in a single CH_2_ group in an aliphatic side chain (butyl vs. pentyl). Such results are encouraging, but in case of sensors, the demonstration of good selectivity in separate solutions is not sufficient, because one needs to see also the response in mixed solutions. This was not measured in the cited paper, and therefore, the mentioned unusual selectivity has not been fully proved. Regrettably, the paper lacked also an explanation for the QCM sensor element’s apparent extraordinarily high sensitivity, which exceeded the manufacturer’s data by orders of magnitude.

The lack of selectivity measurement in mixed solutions is also notable in another MIP QCM sensor work [37]. Even in separate solutions, the selectivity measurements are difficult to interpret. The sensor signal was measured at a single concentration with the analyte and the interferents, respectively. However, since the analyte calibration was linear in the log concentration, not in the concentration itself, the presented interference data cannot be interpreted.

#### 3.6.2. Voltammetric Sensors

Voltammetric sensing may come in many different forms such as cyclic voltammetry, impedance measurement, etc. In these techniques, transport phenomena are measured. Transport selectivity of MIPs may eventually be correlated with the equilibrium binding selectivity, but this is not necessarily always true. Moreover, some of the most selective voltammetric MIP sensors measure the transport rate, or electron transfer rate, of a test substance, such as hexacyanoferrate, and not of the analyte itself. The role of the adsorbed analyte in these cases appears to be the retardation of the test substance’s mass transport or electron transport rate, although sometimes the opposite effect occurs [38]. The mechanism of the underlying phenomenon for such sensors is not well known yet.

Occasionally, the analyte is adsorbed on the MIP-modified electrode in a preconcentration step, and this is followed by the voltammetric measurement of the adsorbed analyte itself. Impressive selectivity has been achieved by such a sensor for metronidazole [39]. Without reducing the merits of this work, one should note that the selectivity of the NIP-modified electrode had not been checked. The sensitivity of the NIP electrode was only three times lower than that of the MIP electrode. Therefore, it would have been interesting to know, if the NIP electrode was not also selective for metronidazole. If it was, then the selectivity of the MIP electrode may not be entirely attributable to imprinting. Such worry is justified because even a bare carbon paste electrode had shown significant selectivity for metronidazole against another nitro-imidazole drug—tinidazole [40].

#### 3.6.3. Potentiometric Sensors

There are many variations of MIP potentiometric sensors [41], and here only one case will be discussed to show some peculiarities of selectivity. A remarkably successful MIP potentiometric sensor for the antidepressant drug citalopram [42] was made by incorporating the MIP nanoparticles into a plasticized PVC membrane. The selectivity of the obtained sensor was measured according to the rules set for such electrodes by IUPAC. It seems, however, that the selectivity values against some ions were greatly underestimated, i.e., the electrode was much more selective than the measured selectivity coefficients would allow. This can be seen from the citalopram calibration line of the electrode, measured in pH 6 phosphate buffer apparently containing 0.1 M NaCl. If the stated mixed solution selectivity coefficient for sodium ions, 0.01, were correct, then the detection limit for citalopram in the buffer should be around 10^−3^ M, not close to 10^−7^ M, as observed in the calibration. On the other hand, the effect of pH was underestimated in the measurement of real urine samples. The calibration lines taken at various pH values show that the detection limit of the sensor was very sensitive to the sample pH. When moving away one pH unit from the optimal pH of 6.0 in either direction, the detection limit increased by one to two orders of magnitude. Since the normal range of urine pH is between 5.6 and 8, using the electrode in unbuffered urine, and close to the detection limit, was not a good choice.

The great pH dependence of the detection limit in this example is also interesting in a more general sense. The electrode measured cations, but its pH sensitivity was not typical for cation selective electrodes. Normally, one would expect the interference due to a cation, such as the proton, to increase with its concentration. In this case, however, an extremum was observed at pH 6. This may be due to the known behavior of the MIPs made for amine templates, showing a binding optimum at about pH 6. It is very likely, that the peculiar pH dependence of the MIP had caused the above-mentioned underestimation of the electrode’s selectivity, since selectivity was measured apparently in unbuffered solutions.

Chiral selectivity was also claimed in this paper, based on the measured logarithmic Nicholsky selectivity coefficient of −0.34. Since the Nicholsky equation is a quasilinear formula, the measured selectivity means that the sensitivity to the R form was about half of the sensitivity to the S template. This level of selectivity is by far not sufficient to measure the S form selectively in the presence of the R form in comparable concentrations.

#### 3.6.4. Optical Sensors

Optical MIP sensors may be based on various phenomena, such as the displacement of a colored or fluorescent compound by the analyte from the MIP. In another format, the MIP may have optically active pendant groups, which change their activity upon interaction with the template or with an interferent. Complex optical phenomena, when combined with the anyway complex competitive adsorption, may elude simple theoretical treatment.

An example showing the difficulties of interpreting the origin of the selectivity of a MIP optical sensor [35] was discussed above. Another example [43] was favorably reviewed by Uzun and Turner [33]. According to the review, Tran et al. [43] “commented in the light of the results observed, that the array format could be used for the practical determination of perfluorooctane sulfonate in water samples even in presence of structurally similar competitors”. However, despite the very original techniques used in the work of Tran et al., the analytical chemist reader may not be fully satisfied. The calibration curve of the sensor and the selectivity data (Figure 4 and Figure 5 of the paper), when taken together, leave the impression that the selectivity against the likely interferent perfluorooctanoic acid, is only about two-fold. An even bigger problem is that the concentration levels of the two compounds, at which selectivity was tested (and at which blank drinking water had been spiked) were 500 times higher than levels considered safe [44]. In conclusion, the paper did not provide enough evidence, that the sensor “could be used for the practical determination of perfluorooctane sulfonate in water samples even in presence of structurally similar competitors”.

The selectivity data of a surface plasmon resonance (SPR) sensor for the organophosphorous pesticide profenofos [45] have been impressive in separate solutions, and have been accordingly discussed in a review on MIP sensors in this field [46]. Measurements in mixed solutions with the likely interferents and determinations in real contaminated samples (not only spiked ones) would still be important.

### 3.7. Membrane Transport

Membrane transport, in general, is a complex phenomenon. MIP membranes [47,48,49] may facilitate the transport of the template across the membrane. Alternatively, they may retard the transport of the template (by strongly binding it), at least until all binding sites across the thickness of the membrane become equilibrated. The template may also indirectly influence mass transport by swelling or shrinking the membrane.

An ideally selective membrane would allow the target compound to pass across the membrane and retain all other compounds. With membranes based on MIPs this is unlikely to happen. Membrane transport is mainly used in industrial technologies, and MIP membranes do not seem to have reached a level yet where they could be applied in industry. In fact, in a 2020 review paper, co-authored by one of the MIP membrane pioneers [50], MIP membranes are not even mentioned.

MIP membranes have also been developed as sensor elements. This application was mentioned above in the section on sensors.

### 3.8. Catalysis

Catalysts increase the rate of thermodynamically feasible, but slow reactions. If the catalyst is used for preparative purpose, in a solution of the substrate only, one does not need substrate selectivity. If, however, the substrate is in a complex mixture, substrate selectivity may be important. Even in this case, selectivity against close analogs of the substrate is not necessary if such analogs are not present. For these reasons, one may not always find information about catalytic selectivity in papers on catalytic MIPs [51,52,53].

Catalytic MIPs are sometimes used as sensor layers, similar to enzyme modified electrodes. In this case, significant selectivity for the analyte, as the substrate, would be needed.

The binding sites of MIPs may also act as microreactors. The goal in this case is not to make the reaction faster, but to favor the generation of one of the possible reaction products. In this sense, one deals also with a kind of selectivity—the selectivity between different reaction routes. This topic is beyond the scope of the present paper.

## 4. Assessment of the Effect of Imprinting on Selectivity

For MIP developers it is a very important question if the imprinting procedure had imparted selectivity to the MIP in comparison with the NIP. It is well known, for example, that the presence of the template during imprinting may influence the polymer morphology and thus also the specific surface area. If the specific surface area of the MIP is higher than that of the NIP, this alone may increase the template binding. However, the increase of the (accessible) surface area will not, by itself, change the selectivity of the polymer. For this reason, a frequently used test is the comparison of the MIP’s selectivity to that of the NIP. As seen above, in different MIP applications, different concepts, requirements, and tests of selectivity may be used. Thus, the improvement of selectivity due to imprinting may also turn out differently, depending on the method of comparison.

Sometimes, however, the test may even be misleading. In MISPE, a typical test is to compare the MIP and the NIP SPE cartridge under “identical” conditions. This includes the use of the same polymer mass in both cartridges. The compared output is the analyte recovery. In this test, the NIP is usually found to be inferior to the MIP, and therefore the NIP’s selectivity is not investigated further. However, the reason for the poor recovery on the NIP is very likely, that due to the lower template adsorption on the NIP, the analyte breakthrough occurs sooner on the NIP cartridge than on the MIP cartridge. A fair comparison would use an accordingly higher NIP mass. Such a comparison might show that the NIP is also sufficiently selective to render the chromatogram clean.

## 5. Conclusions and Recommendations

The characterization of MIP selectivity is a complex task, and its results may not be easily transferable from one kind of application to another, or even from one real sample to another. Selectivity studies of novel MIPs should always be designed by considering

- the expected application;
- the expected concentration range of the target compound(s) (analyte(s));
- the expected interferents and their expected concentrations.

This means that MIP selectivity is best studied in a sufficiently large number of realistic mixtures of target and interferents. Selectivity studies in separate solutions of target compound and interferents, respectively, may be useful in the earliest phase of method development, but the results of this preliminary study may not correctly predict the selectivity in mixtures. In the next phase of development, mixtures should be studied, and these may be synthetic ones at first. A mathematical formula for describing interferent effects may be searched for or selected from the literature, using these mixtures. In the final stages of method development, the mixtures should be real samples, which should be submitted both to the new method, and to a reliable control method for comparison. A sufficient number and a sufficient variety of real samples should be studied to obtain representative results.

One should not miss a check for the origin of selectivity. The fact that a MIP-based (analytical or other) procedure is selective, does not always mean that the observed selectivity is solely, or even at all, due to imprinting.

Comparison with other sorbents should be made under comparable conditions, instead of identical conditions. An example for this, presented above, has been the comparison of the MIP with the NIP in SPE.

## Data Availability

The data presented in this study are available on request from the corresponding author.
